# Identification of genomic alteration and prognosis using pathomics-based artificial intelligence in oral leukoplakia and head and neck squamous cell carcinoma: a multicenter experimental study

**DOI:** 10.1097/JS9.0000000000002077

**Published:** 2024-09-06

**Authors:** Xin-Jia Cai, Chao-Ran Peng, Ying-Ying Cui, Long Li, Ming-Wei Huang, He-Yu Zhang, Jian-Yun Zhang, Tie-Jun Li

**Affiliations:** a Central Laboratory, Peking University School and Hospital of Stomatology; b National Center of Stomatology and National Clinical Research Center for Oral Diseases and National Engineering Research Center of Oral Biomaterials and Digital Medical Devices; c Department of Oral Pathology, Peking University School and Hospital of Stomatology; d Research Unit of Precision Pathologic Diagnosis in Tumors of the Oral and Maxillofacial Regions, Chinese Academy of Medical Sciences (2019RU034); e Department of Oral and Maxillofacial Surgery, Peking University School and Hospital of Stomatology, Beijing; f Hunan Key Laboratory of Oral Health Research, Xiangya Stomatological Hospital, Central South University, Changsha, People’s Republic of China

**Keywords:** artificial intelligence, genomic alteration, head and neck squamous cell carcinoma, oral potentially malignant disorder, prognosis

## Abstract

**Background:**

Loss of chromosome 9p is an important biomarker in the malignant transformation of oral leukoplakia (OLK) to head and neck squamous cell carcinoma (HNSCC), and is associated with the prognosis of HNSCC patients. However, various challenges have prevented 9p loss from being assessed in clinical practice. The objective of this study was to develop a pathomics-based artificial intelligence (AI) model for the rapid and cost-effective prediction of 9p loss (9PLP).

**Materials and methods:**

Three hundred thirty-three OLK cases were retrospectively collected with hematoxylin and eosin (H&E)-stained whole slide images and genomic alteration data from multicenter cohorts to develop the genomic alteration prediction AI model. They were divided into a training dataset (*n*=217), a validation dataset (*n*=93), and an external testing dataset (*n*=23). The latest Transformer method and XGBoost algorithm were combined to develop the 9PLP model. The AI model was further applied and validated in two multicenter HNSCC datasets (*n*=42 and *n*=365, respectively). Moreover, the combination of 9PLP with clinicopathological parameters was used to develop a nomogram model for assessing HNSCC patient prognosis.

**Results:**

9PLP could predict chromosome 9p loss rapidly and effectively using both OLK and HNSCC images, with the area under the curve achieving 0.890 and 0.825, respectively. Furthermore, the predictive model showed high accuracy in HNSCC patient prognosis assessment (the area under the curve was 0.739 for 1-year prediction, 0.705 for 3-year prediction, and 0.691 for 5-year prediction).

**Conclusion:**

To the best of our knowledge, this study developed the first genomic alteration prediction deep learning model in OLK and HNSCC. This novel AI model could predict 9p loss and assess patient prognosis by identifying pathomics features in H&E-stained images with good performance. In the future, the 9PLP model may potentially contribute to better clinical management of OLK and HNSCC.

## Introduction

Highlights

- The first genomic alteration prediction pathomics-based deep learning model (chromosome 9p loss prediction, 9PLP) in oral leukoplakia (OLK) and head and neck squamous cell carcinoma (HNSCC) was developed, combining the latest Transformer algorithm and XGBoost algorithm.
- The combination of the 9PLP model with tumor-lymph nodes-metastasis stage was used to develop a nomogram model for predicting HNSCC patient prognosis.
- The artificial intelligence model showed high accuracy in OLK and HNSCC, with the area under the curve achieving 0.890 and 0.825, respectively, indicating the high clinical potential of the model. The nomogram model also performed well (the area under the curve was 0.739) in predicting prognosis.
- The artificial intelligence model was promising to contribute to better clinical management of OLK and HNSCC.

Head and neck squamous cell carcinoma (HNSCC) is currently the seventh most common malignant tumor worldwide^1^. Despite receiving treatment, patients with locally advanced disease have a poor prognosis, high risk of recurrence, and unsatisfactory overall survival (OS), with a 5-year survival rate of lower than 27%^2^. Therefore, the early prevention and diagnosis of HNSCC are critical. Oral potentially malignant disorders (OPMDs) have been defined by the WHO as oral mucosal abnormalities that are associated with a statistically higher risk of oral cancer development^3^. Oral leukoplakia (OLK) is the most common OPMD^3^, with a global prevalence of 3.4–4.1%^4–6^. Because of the importance of early identification of OLK cases with a high risk of malignant transformation (MT), the specific risk factors associated with OLK MT have been frequently studied to improve patient prognosis. Currently, histopathological grading of oral epithelial dysplasia (OED) is the most common indicator^7^. Molecular biomarkers, especially genetic alterations, have also been investigated. Studies have reported that combining OED grading with molecular biomarkers would result in higher predictive accuracy than using OED alone^8^.

Copy number alteration (CNA) is a type of genetic alteration that has been demonstrated to be associated with OLK prognosis^8–10^. Examples include the loss of chromosomes 9p, 9q, and 13q and the gain of chromosomes 3q and 20p^8,11,12^. Of these, 9p loss is considered the earliest driver event in OLK MT progression^11–14^. Therefore, detecting 9p loss has great clinical value for the early identification of patients with high MT risk. Furthermore, 9p loss is reportedly associated with poor HNSCC patient prognosis and resistance to immune checkpoint blockade (ICB) therapy^14–16^.

However, the expensive costs, heavy tissue demand, lack of equipment, and complex processes associated with traditional molecular testing methods, such as next-generation sequencing (NGS), have limited their application in clinical practice^17^. Because of this, the advantages of using artificial intelligence (AI)-assisted pathology for molecular alteration screening have become apparent. This novel method detects genomic alterations using only slides stained with hematoxylin and eosin (H&E), a simple and inexpensive histological technique. This method has been used in studies in various cancers, including gastrointestinal tract cancer, breast cancer, lung cancer, and prostate cancer, with good prediction efficiency^18–23^. However, such AI models have not yet been developed in OPMDs or HNSCC to the best of our knowledge, although biomarkers including 9p loss have shown great value in prognosis prediction.

In this study, we developed a pathomics-based genomic alteration detection AI model using the latest Transformer algorithm to predict chromosome 9p loss (9PLP model) in OLK cases. 9PLP was then extended to a cohort of HNSCC samples to detect 9p loss. In both OLK and HNSCC, 9PLP was validated in multicenter cohorts. The 9PLP predictive score was further employed to develop an effective nomogram to predict HNSCC survival.

## Materials and methods

### Study design and participants

**Figure 1:**
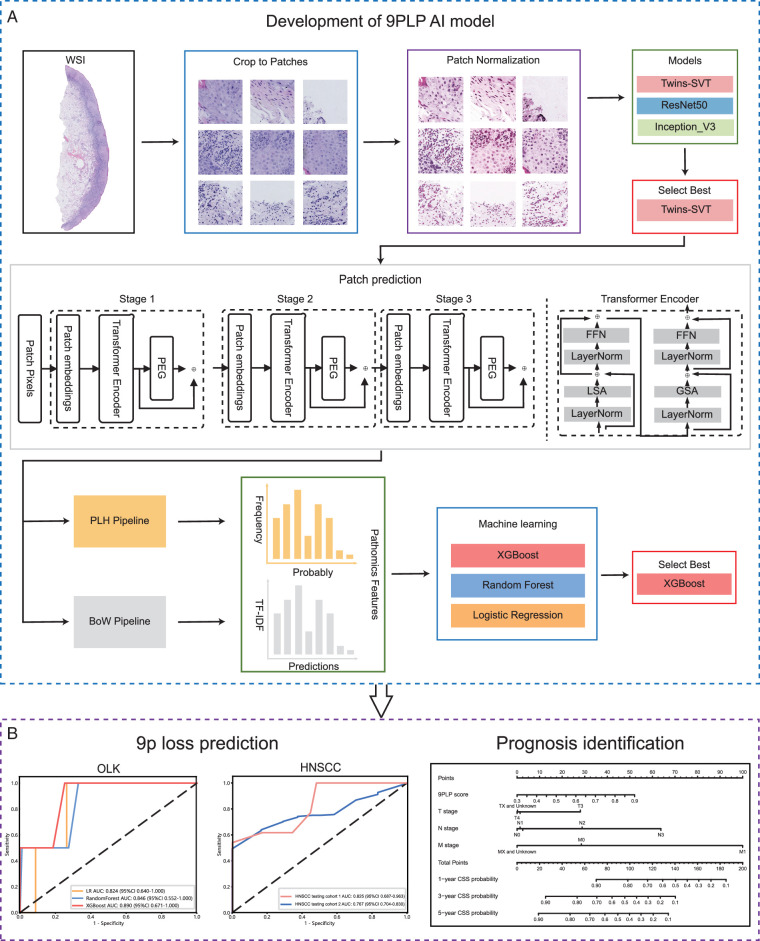
A pathomics-based AI model was developed to predict the genomic alterations in OLK and HNSCC using WSIs. (A) Development of the 9PLP AI model. OLK WSIs were collected and processed, including cropping to patches and normalization. Three deep learning technologies were then used to train the patch level 9p loss prediction model. Two pipelines were employed to generate pathomics features through patch level probabilities fusion. Patient-level prediction results were obtained using machine learning methods with these pathomics features. According to the machine learning method performance results, Twins-SVT and XGBoost were combined as the 9PLP model. (B) 9p loss prediction in OLK and HNSCC, with prognosis evaluation. After being tested in OLK, 9PLP was extended to HNSCC. Receiver operating characteristic (ROC) curve analysis was used to validate the accuracy of the model. The 9PLP score generated by the model was combined with clinicopathological variants to develop a nomogram model for predicting HNSCC patient prognosis. 9PLP, chromosome 9p loss prediction; AUC, area under the ROC curve; AI, artificial intelligence; BoW, Bag of Words; CSS, cancer-specific survival; GSA, global sub-sampled self-attention; HNSCC, head and neck squamous cell carcinoma; LR, Logistic Regression; LSA, locally-grouped self-attention; M stage, metastasis stage; N stage, lymph nodes stage; OLK, oral leukoplakia; PLH, Patch Likelihood Histogram; T stage, tumor stage; TF-IDF, term frequency-inverse document frequency; WSI, whole slide image; XGBoost, eXtreme Gradient Boosting.

In this retrospective study, we developed a pathomics-based AI model to identify genomic alterations in OLK, then extended it to HNSCC, to predict genomic alterations and prognosis (Fig. 1).

**Figure 2:**
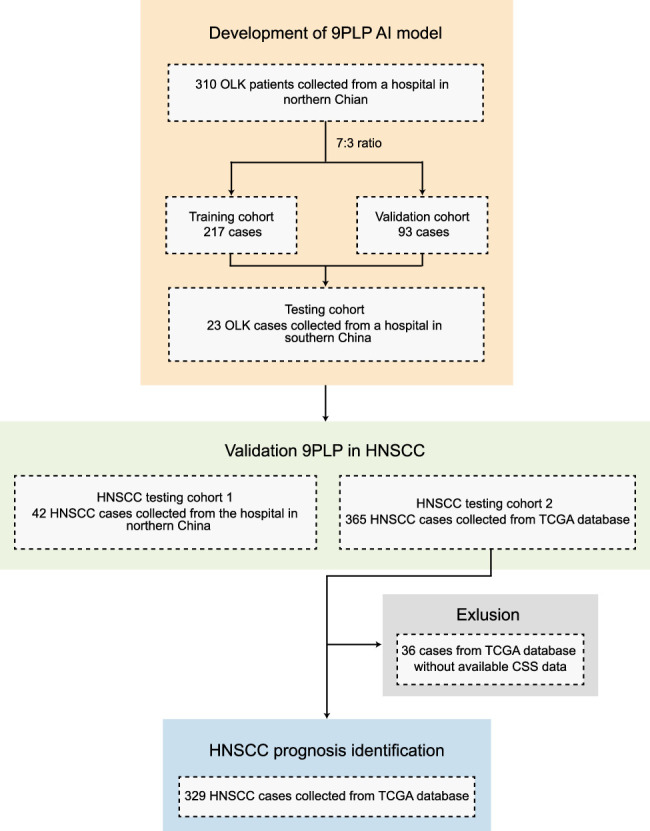
The flow of participant inclusion. 9PLP, chromosome 9p loss prediction; AI, artificial intelligence; CSS, cancer-specific survival; HNSCC, head and neck squamous cell carcinoma; OLK, oral leukoplakia; TCGA, The Cancer Genome Atlas.

Three hundred thirty-three OLK cases with H&E-stained images were collected from multicenter cohorts. Of these, 310 cases were collected from a hospital in northern China from 2014 to 2018, which were then randomly divided into the training cohort (*n*=217) and validation cohort (*n*=93) at a 7:3 ratio. The remaining 23 OLK cases were obtained from a hospital in southern China from 2014 to 2018 as the testing cohort. An AI model was then developed using the H&E-stained images of these 333 OLK cases to predict genomic alteration in this disease. Furthermore, to validate the extension of the AI model for genomic alteration prediction in HNSCC, 42 HNSCC cases and 365 HNSCC cases were respectively collected from the hospital in northern China and The Cancer Genome Atlas (TCGA) database (Supplementary Table 1, Supplemental Digital Content 1, http://links.lww.com/JS9/D416). These were labeled as the extended HNSCC testing cohort 1 and HNSCC testing cohort 2, respectively. The inclusion criteria of the OLK and HNSCC cases are as follows: (a) patients with the diagnosis of OLK or HNSCC by two experienced pathologists according to the criteria presented by the WHO^3,24^, (b) patients with comprehensive clinical and genomic alteration data, (c) patients with a clear and unfaded hematoxylin and eosin (H&E)-stained pathological image. The flow of participant inclusion is presented in Figure 2.

The H&E-stained digital whole slide images (WSIs) included in the training dataset, validation dataset, testing dataset, and HNSCC testing dataset 1 were scanned using the whole slide scanner NanoZoomer (NanoZoomer 2.0-HT, Hamamatsu Photonics), then exported to NDPI style using NDPView2 software (version 2.6.17). The HNSCC testing dataset 2 was downloaded from the TCGA database with SVS style. Baseline data, including age, sex, site of lesions, OED grade, tobacco smoking, and alcohol consumption, were obtained. For HNSCC cases, information about race, tumor stage (T stage), lymph nodes stage (N stage), metastasis stage (M stage), clinical stage, and pathological grade and OS data were also collected. Since unavailable cancer-specific survival (CSS) data for 36 cases, only 329 cases with CSS data were collected. The study was approved by the Institutional Ethical Review Board of the hospital.

### Genomic alteration data

In the previous study^9^, morphology-related laser capture microdissection was used to isolate microcells (~1000 cells) from formalin-fixed paraffin-embedded samples, then low-depth whole-genome sequencing was conducted to detect CNAs, including loss of chromosome 9p. We also downloaded the CNA data of HNSCC cases from the TCGA database for further analysis^11^. As mentioned previously, 9p loss refers to a loss at chromosome 9p irrespective of the length of the deletion^16^. In this study, genomic data were used to develop the 9PLP model.

### Data processing

As described in previous studies^25,26^, we applied a range of data processing methods to improve the quality of input data during model development. All H&E-stained images were de-identified before data processing. Because of the large digital images, WSIs were divided into small segments (512×512 pixels), at a resolution of 0.5 μm/pixel, using a nonoverlapping technique. Segments containing only a white background were excluded, while other segments underwent color normalization using the Macenko method^26^. Z-score normalization was further performed on the red, green, and blue channels to normalize the distribution of image intensities. According to Shorten *et al*.^27^, online techniques, including random cropping and horizontal/vertical flipping, were used for data augmentation to enhance the effectiveness of data and improve the generalization ability of the AI model, avoiding overfitting. For patches in the testing dataset, only normalization was applied.

### Patch level prediction

As described in previous studies^25,26^, our deep learning pipeline contained two prediction procedures, patch-level prediction, and WSI-level prediction, in which multi-instance learning-driven fusion was applied. All WSIs were labeled with binary status, which was linked to the existence of 9p loss or the opposite. Deep learning models were then used to predict the labels and corresponding probabilities for all patches.

Based on previous studies^25,26,28–33^, two networks that were commonly used and are effective in developing pathomics-based AI models were evaluated, including ResNet50, Inception_v3. We also leverage the latest Transformer technology to compare the performance of traditional convolutional neural network (CNN)-based models with those based on Transformer architectures. ResNet and Inception are classic CNN models that generate image features through multiple CNN layers to achieve final classification. As illustrated in Figure 1, Twins-SVT introduces a novel local-global attention mechanism, which is termed Spatially Separable Self-Attention (SSSA), consisting of locally- grouped self-attention, and generates the global fusion of the groups’ attention.

To extend the applicability of the model to cohorts with substantial heterogeneity, we employed transfer learning. This process consists of initializing the model parameters using pretrained weights from the ImageNet dataset and fine-tuning specific layers of the pretrained model to adapt it to our specific task. To enhance generalization, we set the learning rate. In this study, we employed the cosine decay learning rate algorithm as follows:


$$\eta_{t}=\eta_{\min}^{i}+\frac{1}{2}\left(\eta_{\max}^{i}-\eta_{\min}^{i}\right)\left(1+\cos\;\left(\frac{T_{\mathrm{cur}}}{T_{i}}\pi \right)\right)$$

In this notation, η^i^_min_=0 denotes the minimum learning rate, while η^i^_max_=0.01 represents the maximum learning rate and T_i_=30 signifies the number of iteration epochs. Additional hyperparameters were configured as follows: optimizer - SGD, and loss function - softmax cross entropy. Specific hyperparameter settings were: Logistic Regression: max_iter=10; Random Forest: n_estimators=4, max_depth=2, min_samples_split=2; XGBoost: n_estimators=8, max_depth=2, min_child_weight=2.

The performance of the three algorithms was evaluated with several metrics, including the area under the receiver operating characteristic (ROC) curve, accuracy, negative predictive value (NPV), and positive predictive value (PPV). As one of the most popular metrics used to evaluate the performance of AI models, the area under the ROC curve (AUC) reflects the predictive performance comprehensively. The ROC curve shows the false positive rate (FPR) and the true positive rate (TPR) at different thresholds. Other associated formulas are listed below, where true positive (TP) and true negative (TN) refer to the number of correctly classified positive and negative instances while false positive (FP) and false negative (FN) refer to the number of misclassified negative and positive instances, respectively:


$$\mathrm{Specificity}=1-\mathrm{FPR}=1-\frac{\mathrm{FP}}{\mathrm{FP}+\mathrm{TN}}$$

$$\mathrm{Sensitivity}=\mathrm{TPR}=\frac{\mathrm{TP}}{\mathrm{TP}+\mathrm{FN}}$$

$$\mathrm{Accuracy}=\frac{\mathrm{TP}+\mathrm{TN}}{\mathrm{TP}+\mathrm{FN}+\mathrm{FP}+\mathrm{TN}}$$

$$\mathrm{NPV}=\;\frac{\mathrm{TN}}{\mathrm{TN}+\mathrm{FN}}$$

$$\mathrm{PPV}=\;\frac{\mathrm{TP}}{\mathrm{TP}+\mathrm{FP}}$$

### WSI features fusion and signature building

After patch-level prediction, a classifier was applied to aggregate the probabilities of each patch to obtain features at the WSI-level. As described in previous studies^25,26^, two distinct methods, including the Patch Likelihood Histogram (PLH) pipeline and Bag of Words (BoW) pipeline, were developed to obtain the WSI feature. Implementing these pipelines facilitated the integration of the patch-level prediction results into WSI-level features, which were then applied in downstream analyses, including metastasis and survival analysis.

This study combined patch-level predictions, probability histograms, and term frequency-inverse document frequency features to create distinct patient representations as a pathology signature. These features formed the basis for developing a machine learning algorithm for binary status analysis to predict the existence of 9p loss in patients. To visualize these constructed features, the t-Distributed Stochastic Neighbor Embedding (t-SNE) algorithm was employed. Three machine learning models, including Logistic Regression (LR), RandomForest, and eXtreme Gradient Boosting (XGBoost), were examined in WSI-level prediction to identify which performed best based on several metrics, including the AUC, accuracy, negative predictive value, and positive predictive value.

### Extending model application

The model was further investigated in HNSCC test cohorts 1 and 2 to validate if it could be extended to HNSCC patients. The 9PLP score was used in combination with clinicopathological parameters to develop a nomogram model for predicting HNSCC patient prognosis. Univariate Cox regression analysis was used to identify the risk factors for prognosis, while multivariate Cox regression analysis was used to exclude the confounding factors. ROC curve, calibration curve, and decision curve analyses were then employed to estimate the efficiency and clinical application potential of the model.

### Statistical analyses

The analytical tools included Python (version 3.7.12) with the following packages: Pandas 1.2.4, NumPy 1.20.2, PyTorch 1.8.0, Onekey 3.1.3, OpenSlide 1.2.0, SciPy 1.7.3, scikit-learn 1.0.2, and Slideflow 2.1.0. To develop the nomogram model, IBM SPSS Statistics (version 24.0) was used to perform univariate and multivariate Cox regression analyses. ROC curve, calibration curve, and decision curve analyses were performed using R software (version 4.0.5). *P*<0.05 was considered statistically significant.

## Results

### Chromosome 9p loss prediction model development

To develop the model, we randomly divided 310 OLK cases into a training dataset (*n*=217) and a validation dataset (*n*=93) at a 7:3 ratio. Twenty-three OLK cases were also obtained from an external academic medical center as the testing dataset. Table 1 shows the baseline data of the three datasets and Figure 1 displays a flowchart illustrating the general flow of model development.

**Table 1:** Baseline data of training set, validation set, and testing set.

Datasets	Training dataset	Validation dataset	Testing dataset
No.	217	93	23
Median age	55 (45–65)	58 (47–66)	44 (34–50)
Sex			
Female	117 (53.92%)	49 (52.69%)	1 (4.35%)
Male	100 (46.08%)	44 (47.31%)	22 (95.65%)
Site			
Palate	6 (2.76%)	3 (3.23%)	1 (4.35%)
Lip	8 (3.69%)	1 (1.08%)	2 (8.70%)
Gingiva	25 (11.52%)	16 (17.20%)	0
Buccal	66 (30.41%)	37 (39.78%)	10 (43.48%)
Tongue	112 (51.61%)	36 (38.71%)	10 (43.48%)
Histological grade			
Hyperplasia	66 (30.41%)	35 (37.63%)	12 (52.17%)
Mild dysplasia	87 (40.09%)	36 (38.71%)	9 (39.13%)
Moderate dysplasia	43 (19.82%)	12 (12.90%)	2 (8.70%)
Severe dysplasia	21 (9.68%)	10 (10.75%)	0
Tobacco smoking			
No	155 (71.43%)	72 (77.42%)	7 (30.43%)
Yes	62 (28.57%)	21 (22.58%)	16 (69.57%)
Alcohol consumption			
No	176 (81.11%)	75 (80.65%)	11 (47.83%)
Yes	41 (18.89%)	18 (19.35%)	12 (52.17%)

### Patch level performance

**Figure 3:**
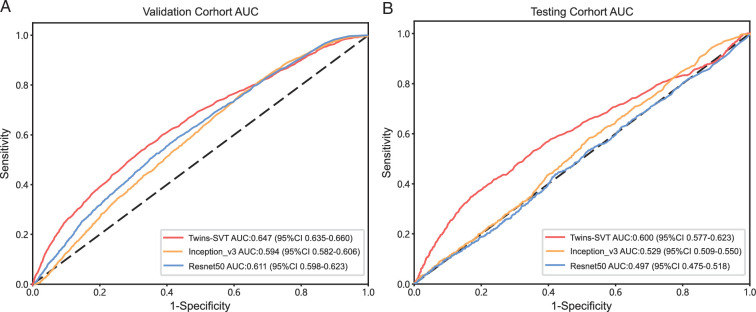
Performances of deep learning models in the validation and testing datasets. (A) Receiver operating characteristic (ROC) curves for each model’s performance in the validation dataset. (B) ROC curves for each model’s performance in the testing dataset. AUC, the area under the ROC curve; CI, confidence interval.

**Figure 4:**
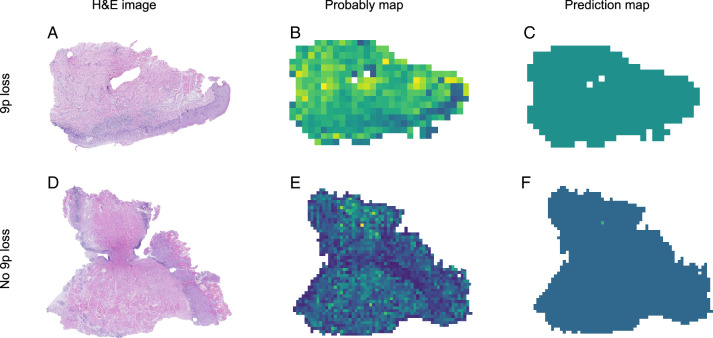
The probably and prediction maps of the genomic alteration prediction model. H&E-stained slides of oral leukoplakia (A, D). Probably maps of the chromosome 9p loss probability for each patch (B, E). Prediction maps of 9p loss in the whole slide images (WSIs) (C, F). For the probably map, a patch color closer to yellow represents a higher predicted probability of 9p loss, while purple represents a lower probability of 9p loss. For the prediction map, a color closer to green represents a higher predicted probability of 9p loss at the WSI-level, while blue represents a lower predicted probability of 9p loss at the WSI-level. H&E, Hematoxylin and eosin.

Three deep learning models, including Twins-SVT, ResNet50, and Inception_v3, were employed to develop the patch-level model. The patch-level model performance data, including the AUC, accuracy, sensitivity, and specificity values, are shown in Supplementary Table 2 (Supplemental Digital Content 2, http://links.lww.com/JS9/D417). Figure 3 shows the ROC curves for each model’s performance in the validation and testing datasets. The results demonstrate that the Twins-SVT model outperformed the CNN-based models in the validation dataset (AUC=0.647, 95% CI: 0.635–0.660) and testing dataset (AUC=0.600, 95% CI: 0.576–0.622). The Twins-SVT model was less prone to overfitting compared with its CNN counterparts. After patch level deep learning, each patch was linked to a corresponding probability. The probabilities of the patches are visualized in the maps shown in Figure 4. The results demonstrated that the Twins-SVT model exhibited high accuracy for predicting chromosome 9p loss at the patch level.

### WSI-level performance

**Figure 5:**
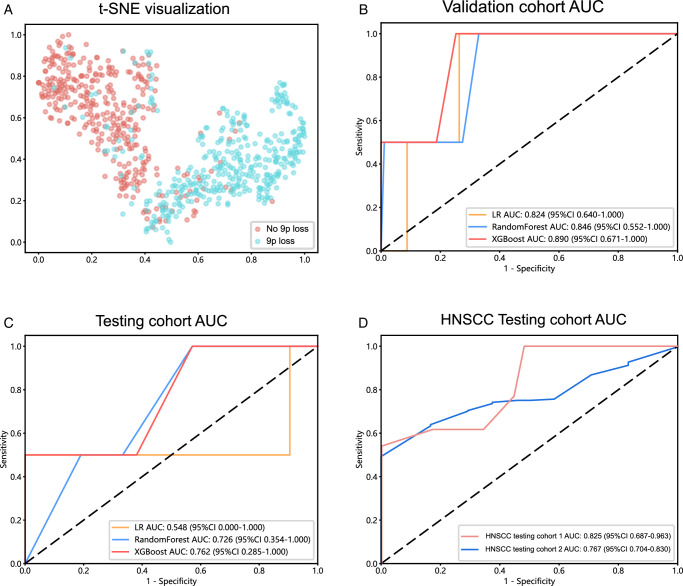
Patient-level prediction performance of the chromosome 9p loss prediction (9PLP) model in oral leukoplakia (OLK) and HNSCC. (A) t-SNE visualization of the patient-level features generated by multi-instance learning. (B) Receiver operating characteristic (ROC) curves in the OLK validation cohort for the three machine learning models. (C) ROC curves in OLK testing cohort for the three machine learning models. (D) 9PLP model performance in HNSCC testing cohort 1 and HNSCC testing cohort 2. AUC, the area under the ROC curve; CI, confidence interval; HNSCC, head and neck squamous cell carcinoma; LR, Logistic Regression; t-SNE, t-Distributed Stochastic Neighbor Embedding; XGBoost, eXtreme Gradient Boosting.

A total of 102 features were aggregated using multi-instance learning (Supplementary Table 3, Supplemental Digital Content 1, http://links.lww.com/JS9/D416) through two distinct methods: PLH pipeline and BoW pipeline. Correlation-based selection was applied to these features. Only one feature from any pair with a Pearson correlation coefficient greater than 0.9 was retained. The t-SNE algorithm was employed to visualize the constructed features, which showed good discrimination for predicting 9p loss (Fig. 5A). These features were then used to train the patient-level prediction model with three machine learning methods, including LR, RandomForest, and XGBoost. The performances of these three models are listed in Supplementary Table 4 (Supplemental Digital Content 2, http://links.lww.com/JS9/D417), while the ROC curves are presented in Figure 5B and C. The results demonstrated that XGBoost had the highest AUC value in the validation dataset (AUC=0.890, 95% CI: 0.671–1.000) and testing dataset (AUC=0.762, 95% CI: 0.285–1.000). Thus, the 9PLP pathomics-based model was finally developed using the Twins-SVT model combined with the XGBoost algorithm.

### Extension of the 9PLP model to HNSCC samples

OLK is the most common OPMD^3,6^ and could malignantly transform into HNSCC with abnormal proliferation of oral epithelium cells. Therefore, our pathomics-based model was further used to predict chromosome 9p loss in HNSCC samples. We collected 42 HNSCC cases from the hospital in northern China as HNSCC testing dataset 1 and 365 HNSCC cases from the TCGA database as HNSCC testing dataset 2. The baseline characteristics of the two datasets are shown in Table 2. As shown in Figure 5D and Supplementary Table 5 (Supplemental Digital Content 2, http://links.lww.com/JS9/D417), we found that the 9PLP model also showed favorable performance in predicting 9p loss in both HNSCC testing dataset 1 (AUC=0.825, 95% CI: 0.687–0.963) and testing dataset 2 (AUC=0.767, 95% CI: 0.704–0.830). These results suggested that the application of 9PLP could be extended from OLK to HNSCC for predicting 9p loss.

**Table 2:** Baseline characteristics of HNSCC testing dataset 1 and HNSCC testing dataset 2.

Datasets	HNSCC testing dataset 1	HNSCC testing dataset 2
Total	42	365
Median age	55.5 (48.25–68)	62 (54–69)
Sex		
Female	12 (28.57%)	107 (29.32%)
Male	30 (71.43%)	258 (70.68%)
Site		
Lip, buccal, gum, and palate	12 (28.57%)	96 (26.30%)
Tongue	26 (61.90%)	103 (28.22%)
Floor of mouth	3 (7.14%)	48 (13.15%)
Pharynx and Larynx	1 (2.38%)	118 (32.33%)
Clinical stage		
stage I	16 (38.10%)	12 (3.29%)
stage II	9 (21.43%)	67 (18.36%)
stage III	5 (11.90%)	77 (21.10%)
stage IV	12 (28.57%)	199 (54.52%)
Unknown	0	10 (2.74%)
Histological grade		
G1	13 (30.10%)	44 (12.05%)
G2	28 (66.67%)	237 (64.93%)
G3	1 (2.38%)	75 (20.55%)
Unknown	0	9 (2.47%)
Tobacco smoking		
No	26 (61.90%)	149(40.82%)
Yes	16 (38.10%)	216(59.18%)
Alcohol consumption		
No	33 (78.57%)	124 (33.97%)
Yes	9 (21.43%)	241 (66.03%)

### The 9PLP score served as a predictive marker of HNSCC patient prognosis

We further explored if the 9PLP score could serve as an indicator of HNSCC patient prognosis because chromosome 9p loss is associated with prognosis in some cancers^14,16,34–37^. No significant association was found between the 9PLP score and HNSCC patient OS according to the univariate Cox regression analysis [hazard ratio (HR)=2.532, 95% CI: 0.799–8.026, *P*=0.114]. However, the CSS of HNSCC patients was significantly associated with the 9PLP score (HR=6.684, 95% CI: 1.607–27.798, *P*=0.009). Notably, the multivariate Cox regression analysis results indicated that the 9PLP score was still a significant indicator of HNSCC prognosis prediction after excluding confounding factors, suggesting that the 9PLP score was associated with poor HNSCC patient CSS (HR=10.265, 95% CI: 2.445–43.093, *P*=0.001). Furthermore, the clinicopathological parameters associated with the CSS of HNSCC patients were identified by univariate and multivariate Cox regression analyses, including T stage, N stage, and M stage. The analyses showed that there was significantly poorer CSS in the HNSCC T3 stage (HR=2.048, 95% CI: 1.182–3.548, *P*=0.011) compared with that in the T1 and T2 stages. Both the N2 stage (HR=2.168, 95% CI: 1.360–3.455, *P*=0.001) and N3 stage (HR=5.522, 95% CI: 1.638–18.610, *P*=0.006) were associated with a poorer prognosis than the N0 stage. The M1 stage (HR=6.796, 95% CI: 1.935–23.862, *P*=0.003) also showed lower CSS than the M0 stage (Table 3).

**Table 3:** Univariate and multivariate Cox regression analyses of prediction probability of 9PLP score and clinicopathological parameters.

Variable	Univariate Cox regression analysis		Multivariate Cox regression analysis	
	HR (95% CI)	*P*	HR (95% CI)	*P*
9p loss	6.684 (1.607–27.798)	0.009	10.265 (2.445–43.093)	0.001
Age (y)	1.000 (0.982–1.019)	0.985		
Sex				
Male	Reference		
Female	0.881 (0.551–1.407)	0.595		
Race				
White	Reference		
Black and African American	1.561 (0.865–2.815)	0.139		
Asian, American Indian, Alaska Native, and Unknown	0.748 (0.303–1.848)	0.529		
T stage				
T1 and T2	Reference		Reference	0.008
T3	1.987 (1.177–3.355)	0.010	2.048 (1.182–3.548)	0.011
T4	1.020 (0.604–1.722)	0.941	0.965 (0.553–1.685)	0.901
Tx and unknown	0.455 (0.061–3.374)	0.441	0.970 (0.032–29.505)	0.986
N stage				
N0	Reference		Reference	
N1	1.175 (0.653–2.114)	0.591	1.037 (0.562–1.912)	0.908
N2	2.203 (1.397–3.475)	0.001	2.168 (1.360–3.455)	0.001
N3	3.900 (1.198–12.693)	0.024	5.522 (1.638–18.610)	0.006
NX and unknown	0.707 (0.170–2.938)	0.633	1.071 (0.146–7.869)	0.946
M stage				
M0	Reference		Reference	
M1	7.339 (2.286–23.561)	0.001	6.796 (1.935–23.862)	0.003
MX and unknown	0.461 (0.114–1.874)	0.279	0.466 (0.064–3.377)	0.450
Clinical stage				
stage I	Reference	0.683		
stage II	0.952 (0.273–3.314)	0.938		
stage III	1.112 (0.326–3.797)	0.865		
stage IV	1.292 (0.405–4.119)	0.665		
Unknown	0.433 (0.045–4.167)	0.469		
Site				
Lip, buccal, gum, and palate	Reference	0.531		
Tongue	1.271 (0.738–2.189)	0.387		
Floor of mouth	1.516 (0.778–2.951)	0.221		
Pharynx and larynx	1.022 (0.591–1.767)	0.939		
Histological grade				
G1	Reference	0.758		
G2	1.205 (0.633–2.294)	0.570		
G3	1.309 (0.638–2.689)	0.463		
GX and Unknown	0.538 (0.069–4.173)	0.553		
Alcohol consumption				
No/ unknown	Reference			
Yes	1.373 (0.870–2.168)	0.173		
Tobacco smoking				
No/ unknown	Reference			
Yes	1.304 (0.853–1.992)	0.220		

9PLP, 9p loss prediction; CI, confidence interval; HR, hazard ratio; M stage, metastasis stage; N stage, lymph nodes stage; T stage, tumor stage.

**Figure 6:**
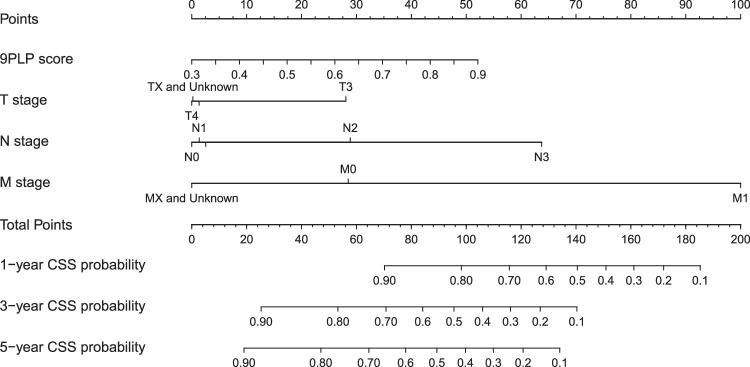
Nomogram prediction model. The 9PLP score, T stage, N stage, and M stage were included and each patient received a total predictive point to identify the 1-year, 3-year, and 5-year CSS rates of head and neck squamous cell carcinoma. 9PLP score, chromosome 9p loss prediction score; CSS, cancer-specific survival; M stage, metastasis stage; N stage, lymph nodes stage; T stage, tumor stage.

**Figure 7:**
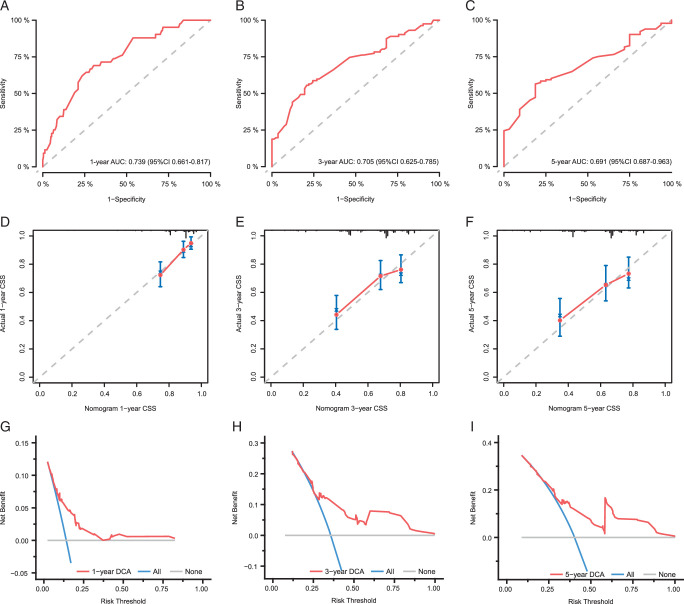
Receiver operating characteristic (ROC) curve, calibration curve, and decision curve analyses of the head and neck squamous cell carcinoma patient prognosis prediction nomogram model. ROC curve analysis and AUC values of the 1-year (A), 3-year (B), and 5-year (C) follow-up. A calibration curve validation for the 1-year (D), 3-year (E), and 5-year (F) follow-up. DCA for the 1-year (G), 3-year (H), and 5-year (I) follow-up. AUC, area under the ROC curve; CI, confidence interval; CSS, cancer-specific survival; DCA, decision curve analysis.

Next, these variables were combined to develop a nomogram model, in which each patient received a total predictive point for CSS prediction. This point could identify the 1-year, 3-year, and 5-year CSS rates. The nomogram model is presented in Figure 6. ROC curve analysis was employed to validate the accuracy of the nomogram model. The AUC values for the prediction of 1-year (AUC=0.739, 95% CI: 0.661–0.817), 3-year (AUC=0.705, 95% CI: 0.625–0.785), and 5-year (AUC=0.691, 95% CI: 0.594–0.788) CSS were obtained, which demonstrated the high prediction efficiency of the model (Fig 7A–C). A calibration curve with bootstrap (500 resample) validation was also applied, as shown in Figure 7D–F. For the 1-year, 3-year, and 5-year follow-up, the predicted line overlapped well with the reference line, especially for the 1-year follow-up. This emphasized the high consistency of the model. Decision curve analysis was used to estimate the clinical net benefit of the model (Fig. 7G–I). The nomogram showed good clinical net benefits for 1-year, 3-year, and 5-year CSS prediction. Both the ROC curve, calibration curve, and decision curve analyses showed the good potential of the 9PLP-based nomogram for predicting HNSCC prognosis.

## Discussion

Previous studies have reported the rate of OLK MT to be 3–10.9%^6,38–40^. Early identification of OLK with high MT risk is important because it is one of the most common OPMDs. However, how to determine which cases can progress to HNSCC remains a challenge^3,7,25^. Analyzing genomic alterations may help overcome this^8^. A large proportion of OLK cases harbor genomic alteration events, including CNAs and gene mutations^8^. CNAs are associated with the evolution of lesions from precancerous to cancerous and occur earlier than histopathological changes^9,10^. Chromosome 9p loss is one of the earliest driver genomic alterations in OLK progression to HNSCC, which has been identified as a potential biomarker to assess the MT risk of OPMDs^11–14^. The role of chromosome 9p loss in OLK MT is associated with immune microenvironment switch and immune escape by suppressing cellular immunity, inhibiting chemokine production, and decreasing T cell infiltration levels^16^. T cell recruitment is suppressed by senescence-associated secretory phenotype decreasing, which is suppressed through cyclin dependent kinase inhibitor 2A (*CDKN2A*, located at chromosome 9p21.3) loss^16,41^. Additionally, loss of RAN binding protein 6 (*RANBP6*, located at 9p13) and interleukin33 (*IL33*, located at 9p13) has been reported to suppress immune cell recruitment while TOP1 binding arginine/serine-rich protein (*TOPORS*, located at 9p13) can suppress NF-κB signaling pathway, causing T cell depletion and immune escape^16^. This immune-exclusive microenvironment caused by chromosome 9p loss not only affects the OLK MT but also might be a potential biomarker of HNSCC patient prognosis^14–16^. Programmed cell death 1 ligand 1 (PD-L1) has been identified as a critical therapeutic target for treating HNSCC^42^. However, only 20% of HNSCC patients would benefit from anti-PD-L1 therapy, and this subgroup is difficult to assess^42^. Chromosome 9p loss could affect the tumor’s immune microenvironment and is associated with resistance to ICB therapy^14,15^. Additionally, the interferon-γ pathway, activated by T cells recognizing the neoantigens of tumor cells, can then activate the JAK-STAT signaling pathway^15^. The key gene Janus kinase 2 (*JAK2*) is located at chromosome 9p24.1 and adjacent to the gene encoding PD-L1^15^. The co-deletion of *JAK2* and *PD-L1* has also been demonstrated to be related to therapeutic resistance^16^.

Notable new molecular technologies have been introduced for decades, including NGS^43,44^. These methods are characterized by high-throughput and increased accuracy, supporting the detection of genomic alterations. However, the implementation of advanced molecular testing methods in routine clinical practice is currently limited for many reasons, including the high cost, lack of infrastructure, requirement of sufficient tissue for testing, and difficulties with interpreting the results^17,45^. Considering the necessity and importance of genomic alteration testing and the limitations of traditional testing methods, AI technology may be an accessible solution for expanding the use of such tests in clinical care. Pathomics-based AI is a promising method for identifying genetic alterations in diseases using only WSI^17^. Such pathology-based AI models have been developed for various cancers, including gastrointestinal tract cancer, breast cancer, lung cancer, and prostate cancer, showing good prediction abilities^18–22^. However, although genomic biomarkers, including 9p loss, have shown value for MT prediction in OPMDs, AI models for genomic alteration detection have not been developed in this disease area.

In this study, we developed a digital pathology-based AI approach that can be used to predict the occurrence of 9p loss in OLK cases by scanning a digital H&E-stained slide without complex molecular testing. To the best of our knowledge, our research was the first to apply pathology-based AI for detecting genomic alterations in OPMDs. Our 9PLP model combined the latest Transformer technology and machine learning model, which provided high predictive efficiency at the patient-level (AUC=0.890, 95% CI: 0.671–1.000). Furthermore, 9PLP utilized weakly supervised multiple instance learning (WS-MIL) methods without manual annotations. Since annotations are marks made by clinicians to identify regions of interest, WS-MIL can substantially reduce clinicians’ workload^46^, considering the huge demand for samples to train an AI model. 9PLP demonstrated unique clinical value for expanding the clinical application of molecular testing for 9p loss in OPMDs. Using 9PLP, clinicians could predict the probability of 9p loss with only an H&E slide. Biopsy specimens excised from OPMD lesions are often too small to perform traditional genetic tests, while an H&E slide is routinely required. Compared with traditional genetic testing, 9PLP is much cheaper, more rapid, and more efficient, allowing the large-scale prediction of 9p loss. Furthermore, 9PLP does not require annotations and is therefore less labor intensive.

As one of the driving genomic events, chromosome 9p loss occurs in the early stage of OLK MT into HNSCC^11,16^, we further explored if 9PLP could be extended to HNSCC. Our results indicated that 9PLP had high specificity and sensitivity for predicting the 9p loss in multicenter cohorts (HNSCC testing dataset 1 AUC=0.825, 95% CI: 0.687–0.963; HNSCC testing dataset 2 AUC=0.767, 95% CI: 0.704–0.830). These findings showed that this model held promise for assisting with the clinical management of HNSCC. Also, it suggests that 9p loss may induce similar histopathological morphology and phenotypes in both OLK and HNSCC, which can be identified by the 9PLP model.

Furthermore, because of the clinical prognosis prediction value of 9p loss in HNSCC, we also used the 9PLP score to establish a prognosis prediction model. Many prognostic indicators have been identified, including the TNM stage and certain biomolecular markers^47^. Although their roles in predicting prognosis are still being studied in more detail, it is clear that combining multiple factors can provide a better prediction performance than using factors individually^47^. Nomogram models are commonly used for predicting cancer patient prognosis because they can integrate various risk factors into a simple numerically estimated model that is practical for clinical decision-making^39^. Such prediction models have been developed for HNSCC, with studies demonstrating that nomograms of combined clinicopathological indicators and biomolecular markers could predict HNSCC prognosis well^48–50^. Therefore, we collected clinicopathological variants and genomic biomarkers to develop a nomogram model with high accuracy and clinical value. Our data suggested that only the TNM stage and 9PLP score were statistically significantly correlated with prognosis in both the univariate and multivariate Cox regression analyses. These factors were both included in our model. The ROC curve, calibration curve, and decision curve analyses further suggested that the model performed well and had high clinical value. These results further emphasized that the 9PLP score is a significant prognostic factor for HNSCC.

Due to the importance of chromosome 9p loss as a prognostic predictive biomarker for OLK and HNSCC, the difficulty of large-scale clinical practice of molecular testing, and the superior predictive performance of the 9PLP, the AI model exhibited promising clinical value, assisting surgeons in predicting important biomarkers, evaluating OLK and HNSCC patient prognosis rapidly and cost-effectively, and subsequently implementing the most suitable treatment^51^. Furthermore, AI models have recently been subjected to clinical trials, thereby providing additional evidence of the potential value and accessibility of AI models in clinical applications^52–54^. However, there are also potential factors that may affect the generalization of such AI models, including different quality control levels of H&E-stained images in various regions, and training models to predict multiple diseases and genomic alterations. These issues are commonly faced by currently developed AI models. In perspective, internationally standardized criteria and larger multicenter cohorts with multiple lesion sites and genomic alterations data may be required to further improve the generalization ability and robustness of the models. As a current research frontier, generative AI may offer a potential solution to these problems through the generation of synthetic datasets and the facilitation of data extraction and organization^55,56^. However, recent research in the HNSCC field has shown that generative AI also has limitations^57^, and further study is needed. Our novel study in the field of pathomics-based molecular prediction AI models in OLK and HNSCC will help more potential technologies, such as generative AI, to be applied in the field of surgery and biomarker prediction.

To the best of our knowledge, this is the first pathomics-based AI model developed to detect genomic alterations in OPMDs. Our work showed that this model was well extended to HNSCC. Furthermore, the model was validated using both OLK and HNSCC multicenter datasets. However, this study still has some limitations. Firstly, we developed the 9PLP model only with OLK cases, the most common OPMD. Studies on other OPMD types are lacking. Secondly, the nomogram model was not validated using an external dataset. Additionally, the experimental design was retrospective. In the future, we will extend the application of the 9PLP and design prospective studies, as well as validate the prognostic prediction nomogram model with more datasets.

## Conclusion

This study developed a 9PLP artificial intelligence platform, which is the first pathomics-based AI model to detect molecular alteration in OPMDs. The model showed high accuracy in the prediction of 9p loss in multicenter datasets and was well extended to HNSCC datasets. Furthermore, an effective nomogram that combined the 9PLP score and clinicopathological parameters was developed to predict the HNSCC patient prognosis. The model demonstrated promising clinical application in the management of OLK and HNSCC.

## Ethical approval

This study was approved by The Institutional Review Board of Peking University School and Hospital of Stomatology (Approval No. PKUSSIRB-202497028).

## Consent

Not relevant to the manuscript due to the retrospective nature of the study.

## Source of funding

This work was supported by the National Nature Science Foundation of China (81671006), the CAMS Innovation Fund for Medical Sciences (2019-I2M-5-038), the Beijing Natural Science Foundation (Z240003), the Postdoctoral Fellowship Program of China Postdoctoral Science Foundation (GZB20240038). The study sponsors had no involvement in the collection, analysis and interpretation of data, the writing of the manuscript, and in the decision to submit the manuscript for publication.

## Author contribution

X.C., C.P., H.Z., J.Z., and T.L.: conceptualization and design; X.C., C.P., Y.C., and J.Z.: data curation; X.C., C.P., Y.C, L.L., and M.H.: formal analysis; T.L.: funding acquisition; Y.C, L.L., and M.H.: investigation; X.C., C.P., H.Z. and T.L.: methodology; H.Z., J.Z., and T.L.: project administration; H.Z., J.Z. and T.L.: resources; X.C. and C.P.: software; H.Z., J.Z., and T.L. supervision; X.C. and C.P.: validation; X.C. and C.P.: visualization; X.C. and C.P.: original draft writing; H.Z., J.Z., and T.L.: manuscript review and edit. All authors read and approve the submission of the manuscript.

## Conflicts of interest disclosure

The authors declare no potential conflicts of interest.

## Research registration unique identifying number (UIN)

Not applicable.

## Guarantor

Tiejun Li, Jianyun Zhang, and Heyu Zhang are the guarantors of this paper.

## Data availability statement

Restrictions are applied to the whole imaging and clinical data of the training and testing sets, which are not publicly available due to patient privacy obligations. All data supporting the findings of this study are available on requests for reasonable academic purposes from the corresponding author T.L.

## Provenance and peer review

Not commissioned, externally peer-reviewed.

## Supplementary Material
