# Supplementary material for: Identification of genomic alteration and prognosis using pathomics-based artificial intelligence in oral leukoplakia and head and neck squamous cell carcinoma: a multicenter experimental study
Source: Int J Surg. 2024 Sep 6;111(1):426–38. doi: 10.1097/JS9.0000000000002077 (PMC11745750; doi:10.1097/JS9.0000000000002077)
Supplement: Supplementary file 2 [file js9-111-0426-s002.pdf]

Supplementary Table 2. Patch level performances of deep learning models.

Model Name	Cohort	AUC (95% CI)	Accuracy	Sensitivity	Specificity	PPV	NPV
Inception_v3	training	0.835 (0.832-0.839)	0.750	0.749	0.750	0.320	0.950
	validation	0.594 (0.582-0.606)	0.405	0.752	0.392	0.045	0.977
	testing	0.529 (0.509-0.550)	0.492	0.579	0.482	0.113	0.909
Resnet50	training	0.895 (0.892-0.898)	0.817	0.794	0.820	0.410	0.962
	validation	0.611 (0.598-0.623)	0.574	0.582	0.574	0.049	0.973
	testing	0.497 (0.475-0.518)	0.566	0.434	0.581	0.106	0.900
Twins-SVT	training	0.648 (0.643-0.652)	0.537	0.687	0.513	0.182	0.913
	validation	0.647 (0.635-0.660)	0.619	0.595	0.620	0.056	0.976
	testing	0.600 (0.577-0.622)	0.731	0.405	0.768	0.167	0.919

AUC, area under the receiver operating characteristics curve; AUC, area under the receiver operating characteristics curve; CI, confidence interval; NPV, negative predictive value; PPV, positive predictive value

Supplementary Table 4. WSI level performance of machine learning models.

Model name	Cohort	AUC (95% CI)	Accuracy	Sensitivity	Specificity	PPV	NPV
LR	training	0.798 (0.698 - 0.898)	0.783	0.708	0.793	0.298	0.956
	validation	0.824 (0.640 - 1.000)	0.731	0.500	0.736	0.040	0.985
	testing	0.548 (0.000 - 1.000)	0.913	0.000	1.000	0.000	0.913
RandomForest	training	0.860 (0.795 - 0.924)	0.793	0.792	0.793	0.322	0.968
	validation	0.846 (0.552 - 1.000)	0.720	0.500	0.725	0.038	0.985
	testing	0.726 (0.354 - 1.000)	0.652	0.500	0.667	0.125	0.933
XGBoost	training	0.888 (0.828 - 0.947)	0.839	0.750	0.850	0.383	0.965
	validation	0.890 (0.671 - 1.000)	0.806	0.500	0.813	0.056	0.987
	testing	0.762 (0.285 - 1.000)	0.913	0.000	1.000	0.000	0.913

AUC, area under the receiver operating characteristics curve; CI, confidence interval; NPV, negative predictive value; PPV, positive predictive value; LR, logistic regression; XGBoost, eXtreme Gradient Boosting.

Supplementary Table 5. Performance of the 9PLP model in HNSCC.

Model name	Cohort	AUC (95% CI)	Accuracy	Sensitivity	Specificity	PPV	NPV
LR	testing 1	0.740 (0.560 - 0.920)	0.786	0.462	0.931	0.750	0.794
	testing 2	0.742 (0.670 - 0.814)	0.600	0.581	0.875	0.985	0.128
RandomForest	testing 1	0.780 (0.614 - 0.946)	0.810	0.385	1.000	1.000	0.784
	testing 2	0.694 (0.593 - 0.795)	0.682	0.680	0.708	0.971	0.135
XGBoost	testing 1	0.825 (0.687 - 0.963)	0.810	0.385	1.000	1.000	0.784
	testing 2	0.767 (0.704 - 0.830)	0.167	0.109	1.000	1.000	0.073

AUC, area under the receiver operating characteristics curve; CI, confidence interval; NPV, negative predictive value; PPV, positive predictive value; LR, Logistic Regression; XGBoost, eXtreme Gradient Boosting.
